# TRP Channels Mediated Pathological Ca^2+^-Handling and Spontaneous Ectopy

**DOI:** 10.3389/fcvm.2019.00083

**Published:** 2019-06-20

**Authors:** Martin Ezeani

**Affiliations:** Faculty of Medicine, Nursing and Health Sciences, Alfred Hospital, Monash University, Melbourne, VIC, Australia

**Keywords:** TRP channels, SR Ca^2+^-ATPase, NCX, ectopy, Ca^2+^-handling

## Abstract

Ion channel biology offers great opportunity in identifying and learning about cardiac pathophysiology mechanisms. The discovery of transient receptor potential (TRP) channels is an add-on to the opportunity. Interacting with numerous signaling pathways, being activated multimodally, and having prescribed signatures underlining acute hemodynamic control and cardiac remodeling, TRP channels regulate cardiac pathophysiology. Impaired Ca^2+^-handling cause contractile abnormality. Modulation of intracellular Ca^2+^ concentration ([Ca^2+^]_i_) is a major part of Ca^2+^-handling processes in cardiac pathophysiology. TRP channels including TRPM4 regulate [Ca^2+^]_i_, Ca^2+^-handling and cardiac contractility. The channels modulate flux of divalent cations, such as Ca^2+^ during Ca^2+^-handling and cardiac contractility. Seminal works implicate TRPM4 and TRPC families in intracellular Ca^2+^ homeostasis. Defective Ca^2+^-homeostasis through TRP channels interaction with Ca^2+^-dependent regulatory proteins such as sodium calcium exchanger (NCX) results in abnormal Ca^2+^ handling, contractile dysfunction and in spontaneous ectopy. This review provides insight into TRP channels mediated pathological Ca^2+^-handling and spontaneous ectopy.

## Introduction

Ca^2+^ flux at organellar levels govern excitation and contraction coupling. The ionic movement is important. During action potential propagation, cell membranes depolarize, and Ca^2+^ enters into the cells. L-type calcium channel passes the Ca^2+^ influx as inward Ca^2+^ current (*I*Ca,_L_), which contributes to the shape of action potential plateau. The Ca^2+^ entry initiates Ca^2+^ release from the sarcoplasmic reticulum (SR) by activation of ryanodine receptors (RyRs), thereby elevating [Ca^2+^]_i_ in the so-called Ca^2+^-activated Ca^2+^ release. The raise in the [Ca^2+^]_i_, activated by a variety of ways, is a known mechanism of cell signaling. It enables Ca^2+^ to bind to contractile machineries such as myofilament protein and troponin C, and initiate contraction. The raise in the [Ca^2+^]_i_ contrarily declines in relaxation. Intracellular Ca^2+^ is pumped from the SR by sarcoplasmic reticulum calcium pump (SERCA), and sarcolemmal efflux through sodium calcium exchanger (NCX), with little contribution by the SERCA during relaxation (1). This results in a decline of [Ca^2+^]_i_, allowing Ca^2+^ to dissociate from troponin for relaxation to occur at diastole. While decline in [Ca^2+^]_i_ through Ca^2+^ efflux leads to relaxation, elevation in [Ca^2+^]_i_ through Ca^2+^ influx leads to contraction. The movement of monovalent and divalent cations through the channels is crucial to cardiac excitation and contraction coupling, and in this review, considerations are given to TRPC and TRPM channels in this process.

The process is impaired in contractile abnormality (2) and in general in heart diseases. Abnormal cardiac performance, which characterizes heart failure, cardiomyopathy, and arrhythmia, occurs through impaired Ca^2+^-homeostasis. Compared with non-failing cardiomyocytes, failing cardiomyocytes SR Ca^2+^ re-uptake was reduced with no significant changes in the rate of Ca^2+^ efflux by NCX (3). Frequency-dependent increases in SR Ca^2+^ load, that was associated with a decline in contractile tone at high heart rates, was absent in failing myocardium (4). In addition, it has been demonstrated at single cell levels that alteration in Ca^2+^-handling occurred through abnormal Ca^2+^-homeostasis and increase in [Ca^2+^]_i_ in familial hypertrophic cardiomyopathy disease (5). The impaired Ca^2+^-homeostasis may be due to translational and transcriptional changes at the level of expression of the Ca^2+^-regulatory proteins. Chronic heart failure patients had abnormal changes in the levels of Ca^2+^-regulatory proteins, such as the SR Ca^2+^-ATPase (6). Together, impaired Ca^2+^-homeostasis and altered Ca^2+^-regulatory proteins orchestrate abnormal Ca^2+^-handling. The mechanisms responsible for this are not completely elucidated.

Tremendous efforts at understanding the process is ongoing, but it is hampered by the complicated nature of changes in [Ca^2+^]_i_. Subcellular Ca^2+^ concentration is compartmentalized, and [Ca^2+^]_i_ varies tremendously. Furthermore, intracellular Ca^2+^ homeostasis can be regulated by local signaling processes within restricted space, rather than by just changes in the global cytosolic [Ca^2+^]_i_. With the complexities, it is difficult to completely define distinct Ca^2+^ homeostasis together with their [Ca^2+^]_i_ biological networks, and their roles. This may have implied that sources that control subcellular Ca^2+^ are just too numerous and are yet to be defined completely. This review discusses some transient receptor potential (TRP) channels seminal works in pathologic Ca^2+^-handling and spontaneous ectopy.

## Regulation of [CA^2+^]_i_ and Ca^2+^-Handling

**Figure 1:**
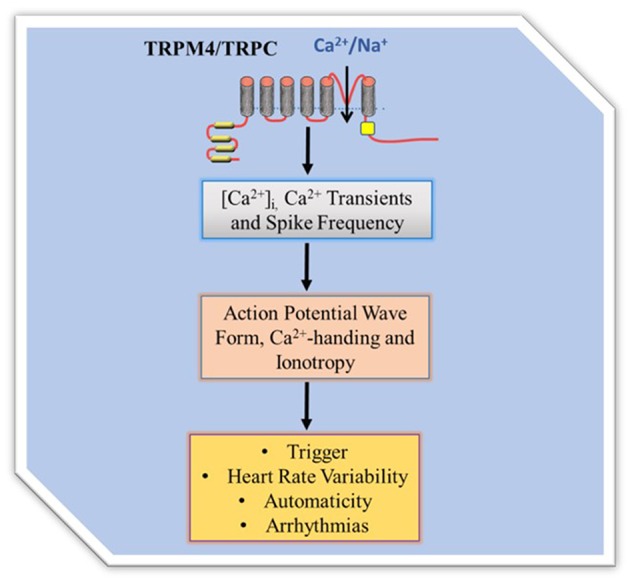
TRP channel regulation of intracellular Ca^2+^ homeostasis. TRPM4 mediates membrane depolarization dependent Ca^2+^ entry while TRPC mediates permeability dependent Ca ^2+^ entry. The Ca^2+^ influx directly and/or indirectly contributes to Ca^2+^ transient to regulate Ca^2+^-handling, action potential wave form and contractility. Since TRPC are Na^+^/Ca^2+^ permeable they can directly contribute Ca^2+^ entry while the Ca^2+^ non permeable TRPM4 regulates Ca^2+^entry by modification of the membrane potential and thus driving force.

Regulation of [CA^2+^]_i_, that is part of CA^2+^ homeostasis, and governs Ca^2+^-handling in excitation and contraction coupling is an important process of cardiac contractile performance. Cardiac contractility is the intrinsic ability of the myocardium to contract. Readily occurrence of contraction depends on incremental degrees of binding between myosin (thick) and actin (thin) filaments, when the [Ca^2+^]_i_ is elevated. Factors that promote Ca^2+^-handling and contractility do so by inducing increases in the [Ca^2+^]_i_. The heart is unable to contract in too little Ca^2+^ concentration (small Ca^2+^ transient amplitude) and unable to relax in too much Ca^2+^ concentration (large Ca^2+^ transient amplitude), indicating negative and positive inotropy, respectively. TRP channels as cell membrane ion channel fall into a category and were first identified in impaired *Drosophila* visual adaption, where photoreceptors carrying TRP gene mutations showed a transient voltage response to continuous light (7, 8). Twenty eight mammalian TRP genes have been identified to date and have fallen into six related protein families. Most of them appear to be multimodally gated and interact with multiple signaling pathways and show pertinent trait to cardiac remodeling. While some are voltage dependent others are not, and they regulate a variety of cell functions such as, apoptosis, thermo regulation, cell viability and proliferation, and renal Ca^2+^ absorption. TRP channels are relatively non-selective permeable cation channels. As such, TRP channels permeate Na^+^, Ca^2+^, and Mg^2+^, and thus regulate intracellular ionic concentrations, including [Ca^2+^]_i_. Seminal works (9–13) implicate some TRPM4 and TRPC channels in [Ca^2+^]_i_ variability. The channels, therefore, modulate Ca^2+^ transients, inotropy, and action potential wave form (Figure 1).

Transient receptor potential melastatin (TRPM) is a family of TRP ion channels, that consists of eight different subfamilies (TRPM1-TRPM8). TRPM4 subfamily while permeable to Na^+^ is activated by [Ca^2+^]_i_, in Ca^2+^-induced Ca^2+^ release process and modulates inotropic β-adrenergic effects on ventricular heart muscle by increasing Ca^2+^ transients amplitude (11). The activation process is important because it contributes to Ca^2+^ transients. Ca^2+^-handling, excitation-contraction coupling and action potential wave forms depend on Ca^2+^ transients, proofing that the [Ca^2+^]_i_ activation and inotropic β-adrenergic effects of TRPM4 are both important in contractility. TRPM4 inhibition altered action potential wave form and reduced action potential duration (13). More so, it has been proposed that TRPM4 channel activity might couple to *I*Ca,_L_ functional activity in elevating [Ca^2+^]_i_. Trpm4^−/−^ ventricular myocytes had fast repolarization as a result of enhanced driving force of *I*Ca,_L_ for Ca^2+^ entry (11). Therefore, it appears TRPM4 regulates action potential adaptation and duration, and underpins voltage-gated Ca^2+^ channel Ca^2+^ influx that couples its activity and that of *I*Ca,_L_ in the so called Ca^2+^-induce Ca^2+^ release that promote contractility.

In addition to TRPM4, TRP canonical (TRPC) is another family of TRP channels. It consists of seven subfamilies (TRPC1-TRPC7). TRPCs are also permeable to but activated by Ca^2+^, and may be important in Ca^2+^-handling. TRPC6 promoter gene had two conserved nuclear factor of activated T-cell transcriptional factor (NFAT) consensus sites (14) and NFAT is calcium-dependent regulatory transcriptional factor. Therefore, TRPC6 is an intracellular Ca^2+^ signaling effector. Increased TRPC1/TRPC4 expression underscores elevated SR Ca^2+^ content in right ventricular hypertrophied cardiomyocytes during monocrotaline exposure (14). Transgenic mice with inhibition of TRPC3/6/7 and TRPC1/4/5 subfamilies had membrane Ca^2+^ leak in pathological hypertrophy following either activation of neuroendocrine (phenylephrine and angiotensin II (Ang II) infusion) or pressure overload induction (15). TRPC channels as cation-selective channels can produce pathological cardiac growth of adult myocytes through Ca^2+^ influx and calcineurin activation, that alter Ca^2+^-handling and Ca^2+^-dependent signaling.

Although it is possible that TRPC/Ca^2+^/calcineurin/NFAT signaling loop might regulate Ca^2+^-handling in diseases, how the signaling loop couples to [Ca^2+^]_i_ and L-type Ca^2+^ channel activity is completely unknown. It appears in smooth muscle that entry of cations through a receptor operated TRPC6 caused membrane depolarization and consequent functional activity of L-type Ca^2+^ channels, Ca^2+^ influx, and smooth muscle contraction. However, whether this role is associated with ryanodine and NFAT is not known. Given that TRPC6 can operate as receptor-activated cation channels, which increases [Ca^2+^]_i_ by Ca^2+^ entry across plasma membrane and/or by release of Ca^2+^ from intracellular stores such as the endoplasmic reticulum, TRPC6 contributes to Ca^2+^-handling and [Ca^2+^]_i_. The contribution can also be deduced on store-operation molecular standpoint. This is since store-operated and receptor-operated channels can come from the same proteins of the same family of TRP channels.

Nonetheless, TRP channels store operated and receptor operated calcium entry (SOCE) and (ROCE) are less clear. Discovery has it that stromal interaction molecule 1 (STIM1) and Orai 1 are mediators of SOCE. Fast Ca^2+^-dependent inactivation kinetics of STIM1 current is known to be critically dependent on cytosolic Ca^2+^ levels, which also regulates native SOCE and ROCE currents, through changes in the [Ca^2+^]_i_, similarly to that of TRP channel currents. Whether TRP channels kinetics are associated to SOCE and ROCE, is not elucidated. Native store-operated and receptor-operated complexes stoichiometry, if distinct from hetero/homo multimer variants Orai or TRP is also not known. Regardless, STIM1 indirectly activates TRPC3/6, but not TRPC7 (16), and TRPC1/4/5 directly bind with STIM1 to activate SOCE (17), and in lipid raft domains, TRPC channels co-localize with STIM1 and Orai (18). There may be a better understanding of TRP channels SOCE attribute arguably in micro-domains (19). In fact, it is known that the Ca^2+^-activated signaling effectors are either in direct proximity or attached to Ca^2+^ entry channels in compartmentalized micro-domains (20). Dissociation of TRPC1 from Cav1, following Ca^2+^-store depletion is an important process in the activation of TRPC1-SOCE (20). Expression of TRPC channels and Ca^2+^ influx through TRPC within micro-domains affects contractility reserve and contributes to cardiac Ca^2+^ cycling. Taken together: (1) direct effects of TRP channels and receptor operated calcium entry mechanisms on [Ca^2+^]_i_ and Ca^2+^-handling may be questioned. (2) It is more likely that TRP channels regulate the process through effects on micro-domains or through direct interaction with Ca^2+^-dependent regulatory proteins. (3) It is noted that STIM1 is a Ca^2+^ sensor that relays Ca^2+^ load of the endoplasmic reticulum to store operated channels and may navigate some of the TRP channels into micro-domains to govern their roles in cardiovascular diseases through calcium signaling effectors.

In addition to the myocardial TRPC channels, atrial endocardial TRPC-6 channel has been identified to be crucial in TRPC-6-dependent paracrine factors that regulate the amplitude of myocardial Ca^2+^ transients through mechanotransduction. Mechanical stretch is a determinant of atrial function such as contractility and TRPC-6 is a stretch responsive cation channel that governs mechanotransduction. To understand the roles of stretched-atrial endocardium, associated with physiological conditions and arrhythmogenesis Nikolova-Krstevski et al., tested the consequences of stretch-induced endocardial TRPC-6 activation on myocardial function and hypothesized that endocardial TRPC-6 is required for mechanical stretch responses in the atrium (21). Findings from this investigation, demonstrate that, in acute stretch, Ca^2+^-mediating function of TRPC-6 was consequentially enhanced via [Ca^2+^]i, and endocardial TRPC-6 regulatory feedback protects against this stretch-induced myocardial Ca^2+^ overload process, whereas in chronic stretch, reduced protein expression of endocardial TRPC-6 and irregular Ca^2+^ transient cyclic events may lead to enhanced susceptibility to arrhythmia. To understand the Ca^2+^ transient mechano feedback response, atrial endocardial-myocardial (cell-cell) communication/signaling was investigated in cultured human-induced pluripotent stem cell-derived cardiomyocytes before and after the addition of media collected from non-stretched and stretched atrial endocardial cells from porcine. The investigation revealed alteration of Ca^2+^ transient in human-induced pluripotent stem cell-derived cardiomyocytes with non-stretched but not with stretched atrial endocardial cells (21). The finding suggests that TRPC-6 dependent stretch-induced mechanotransduction can induce changes in global [Ca^2+^]_I_ that modulate intrinsic atrial endocardial function, such as contractility, as well as affecting myocardial function, through endothelium secreting factors such as endothelin-1, nitric oxide, prostacyclin, and angiotensin II (21).

## Mechanisms of Regulation of [CA^2+^]_i_ and CA^2+^-Handling

The question now remains how does the channels regulate [Ca^2+^]_i_, and thus control Ca^2+^-handling and contractile tone? The mechanisms of TRP channel mediated Ca^2+^ handling remain largely unknown. Ca^2+^-dependent regulatory proteins such as NCX and SERCA regulate Ca^2+^-handling and cardiac contractility, and the TRP channels interact directly with NCX and SERCA. Functional and physical interaction of TRPC channels with NCX proteins is a novel principle behind TRPC-mediated increase in intracellular Ca^2+^ signaling. To assess for the existence of a native TRPC3/NCX1 signaling complex in rat cardiac myocytes, previously identified in HEK 293 cells, the Eder et al conducted reciprocal co-immunoprecipitation to detect TRPC3 and NCX1 interaction by immunoprecipiting solubilized proteins of crude adult rat cardiomyocyte membrane fractions with either anti-TRPC3 or anti-NCX (22). This demonstrated significant TRPC3 and NCX1 co-localization. Glutathione S-transferase pulldown experiment consistently replicated the native TRPC3/NCX1 signaling complex within the cardiomyocyte. To understand the functional consequences of this interaction, phpspholipase C (PLC) was first stimulated in the myocytes, and because TRPC3 is a key component of PLC-dependent Ca^2+^ signaling, the cardiomyocytes were then transiently transfected with TRPC3 N-terminal fragment to exert dominant negative effects on TRPC3. The activation of PLC promoted reverse mode NCX1-mediated Ca^2+^ entry, as identified, through [Ca^2+^]_i_ measurement, that was regulated by TRPC3-mediated Na^+^ loading in the myocytes through angiotensin-induced activation of the G protein Gq-PLC pathway (22). Measurement of this NCX-mediated cellular Ca^2+^ signals in the cells expressing dominant negative TRPC3 revealed significant reduction in Ca^2+^ signals, illustrating the idea of TRPC3-dependent Na^+^ loading, as part of cardiac NCX operation (22).

PLC dependently recruited TRPC3-NCX1 complex into the plasma membrane and regulated Ca^2+^ homeostasis in rat cardiomyocytes (19, 22). TRPC3 channel may be a major component of PLC-dependent activation of the Ca^2+^ calcineurin-NFAT signaling pathway, a pathological cardiac hypertrophy pathway, in which NCX1 can mediate Ca^2+^ influx by reverse mode and contribute to Ca^2+^ transients and action potential wave forms. Together, TRPC3 may be an important component of NCX regulation of Ca^2+^-handling. Furthermore, SERCA is an ATP-dependent Ca^2+^ pump located in the SR membrane. It appears silencing SR Ca^2+^-ATPase (SERCA)2 with small interfering RNA (siRNA) increases TRPC levels (23), as a compensatory mechanism for SERCA Ca^2+^ pump. While colocalizing on the skin of Darier's disease patient on immunostaining, TRPC1 had increased immunoreactivity whereas, SERCA2 had reduced immunoreactivity, suggesting reciprocal activity of TRPC1 and SERCA2 (23). Western blots performed on the skin from the DD patient and immunolocalization performed on skin samples from SERCA2^+/+^ and SERCA2^+/−^ mice showed consistent reciprocal expression of TRPC1 and SERCA2 (23). The DD patients as well as SERCA2^+/−^ mice have only one normal copy of the gene encoding SERCA2. To understand the association of the reciprocal expression, a human keratinocyte (HaCaT) cell line was employed. The keratinocytes from DD patients have also one normal copy of SERCA2. Single copy of the gene is known to be inadequate, to appropriately account for proper amount of SERCA2, leading to changes in Ca^2+^ signaling in the cells. To examine this SERCA2 reduction in function, HaCaT cells were treated with adenovirus encoding either SERCA2-siRNA or control-siRNA and Ca^2+^ imaging was performed on the cells upon stimulation with thapsigargin, to deplete endoplasmic reticulum Ca^2+^ (23). Addition of thapsigargin in HaCaT cells Ca^2+^-containing media, caused an increase in global [Ca^2+^]_i_. Whereas this increase was high in HaCaT cells expressing SERCA2-siRNA, no increase in control-siRNA was documented (23). Consistent with the reciprocal expression of TRPC1 and SERCA2, release of Ca^2+^ from the internal stores upon the thapsigargin addition was low in cells overexpressing SERCA2-siRNA, whereas no changes were seen in TRPC1-overexpressing cells (23).

**Figure 2:**
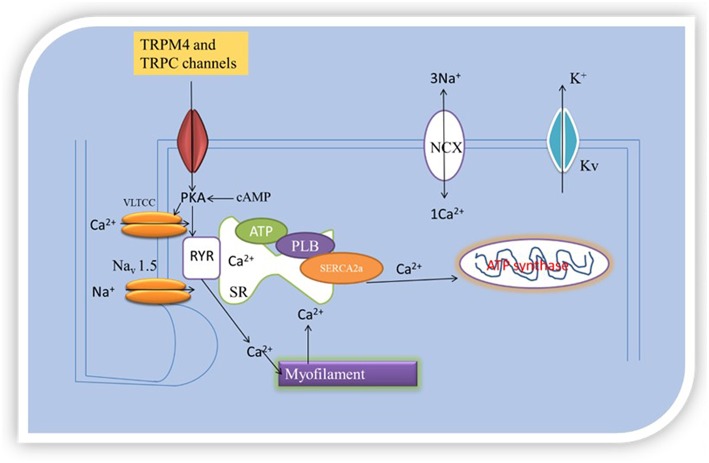
Oganellar Mechanisms of TRP channels mediated pathologic Ca^2+^ handling. Reduced levels of [Ca^2+^]_i_ means that the heart cannot contract and pump blood while high levels of [Ca^2+^]_i_ means that the heart cannot relax. TRPM4 and TRPC interact with Ca^2+^-dependent regulatory proteins (SERCA2a and NCX). How this interaction occurs either reduces or boosts excitation and contraction coupling (EC) by either reducing or increasing [Ca^2+^]_i_. EC is initiated by excitation of the cell membrane which activates Navl.5 leading to the opening of VLTCC. VLTCC opening allows passage of inward Ca^2+^ current that triggers opening of ryanodine receptor 2 (RyR2) channels by a Ca^2+^- induced Ca^2+^ release process. The Ca^2+^-induced Ca^2+^ release leads to coordinated release of sarcoplasmic reticulum (SR) Ca^2+^ caused by PLB phosphorylation. Intracellular free Ca^2+^ concentration become high, activating the myofilaments after binding with troponin C. TRPM4 and TRPC mediated Ca^2+^ entry can couple to this process of elevation in [Ca^2+^]_i_ to cause contraction. This process is part of pathologic Ca^2+^-handling.

Accordingly, SERCA2 silencing was followed by increased transcription of NCX, TRPC4, and TRPC5 in cardiac myocytes (24). Neonatal rat and chicken embryo cardiac myocytes expressing SERCA2-siRNA endogenously had reduced expression of SERCA2 as demonstrated by immunostaining, western blotting, real-time RT-PCR, and microscopy (24). The functional effects of the reduced SERCA2 expression was tested on Ca^2+^ signaling in rat myocytes by measuring Ca^2+^ transients, and *I*Ca,_L_ in neonatal rat myocytes under voltage clamp protocol. Whereas, there was reduced Ca^2+^ transient amplitude (i.e., reduced sarcoplasmic reticulum Ca^2+^ load) in the myocytes subjected to SERCA2-siRNA, there was no differences in *I*Ca,_L_ under the voltage clamp protocol tested. However, the myocytes were shown to have the ability to initiate transient elevations in cytosolic Ca^2+^ upon membrane stimulation, in compensatory mechanisms. To understand this process, the presence or absence of Na^+^ to examine the role of NCX to cytosolic Ca^2+^ removal under voltage-clamp depolarizations, and Ba^2+^ influx measurement in fura-2-loaded myocytes treated with thapsigargin to block SERCA pump activity to examine the role of TRPC channels, in another experiment, were together, employed to access the impact of SERCA2-siRNA on NCX, TRPC channels and Ca^2+^ signaling. This investigation revealed that reduced SERCA2 expression was associated with an elevation in TRPC expression and activity, as well as increased NCX expression and activity, that together may boost and normalize for reduced sarcoplasmic reticulum Ca^2+^ signaling following SERCA2 depletion (24). This mechanism was attributed to NCX and TRPC transcription, as well as, the upregulation of other transcriptional factors such as stimulating protein 1, myocyte enhancer factor 2, and nuclear factor activated T-cell, cytoplasmic 4 (24). In application of this finding, SR releases 70–80% of the Ca^2+^ needed for contraction into the cytosol. SERCA, NCX, and ATP-dependent Ca^2+^pumps remove Ca^2+^ from the cytosol. If reduction in expression of SERCA2, which can directly affect SERCA/Ca^2+^ pump, inversely increases NCX and TRPC levels, then TRPC and NCX govern [Ca^2+^]_i_, excitation- contraction coupling and inotropy, because TRPC and NCX operated Ca^2+^ homeostasis will compensate for SERCA Ca^2+^ pump. Figure 2 illustrates the organellar TRP channels and Ca^2+^-dependent regulatory proteins regulation of Ca^2+^-handling.

Further support is delineated from the study of Oslon's group in 2006. It appears from the study that TRPC6-calcineurin-NFAT pathway, inversely, regulates the expression of SERCA2 (14), suggesting reciprocal relationship between the channel and the protein/gene. What is not known is whether downregulation of TRPC4/5 would upregulate SERCA2 expression in reverse. Understanding the precision of this relationship is pertinent and vital. It is pertinent, since the Seth et al., Eder et al., and the Pani et al. studies highlight novel association between TRP channels and Ca^2+^-dependent regulatory proteins that governs [Ca^2+^]_i_. It is vital since Ca^2+^-dependent regulatory proteins that associate with the TRP channels are potential therapeutic targets for Ca^2+^ -mishandling (25), and Ca^2+^-dependent regulatory gene therapy approaches are being understood to increase myocardial SERCA2a in heart failure contractile abnormality (26). This in part emphasis the importance of this review in development of therapeutic agents that can benefit the heart in contractile dysfunction beyond short-term.

For instance, it is known that Trpm4^−/−^ mice and their control littermate had no differences in cardiac contractile parameters under basal conditions, but infusion of ≤300 ng/kg·min isoprenaline resulted in robust increases (11). This is an observation of incremental physiological contractile activity, which may be rather beneficial in heart failure, cardiomyopathy and arrhythmias, characterized by diminished contractile tone. Thus, genetic downregulation of TRPM4 proteins might be a novel therapeutic approach. Trpm4^−/−^ mice with ischaemic heart failure had improved survival rate and enhanced β-adrenergic cardiac response, indicating better contractile performance (27). Together, TRP channels have pleiotropic roles in the heart (28), and are part of excellent potential targets in gene therapy approach being appreciated to increase myocardial SERCA2a expression in heart failure contractile abnormality.

Given that SERCA2a regulates Ca^2+^ homeostasis, it has impact on systolic and diastolic functions. An elegant approach to improve left ventricular systolic function can be through activation of cardiac myosin, as well. Besides conventional inotropic agents that modulate Ca^2+^ homeostasis in the myocardium, molecular motor (myosin) and sarcomeric scaffolding, and energetic agents referred to as cardiac myotropes and mitotropes, respectively, have been proposed as pharmacological agents that improve myocardial performance (29). The framework of this proposal is to better understand the clinical approaches of agents that ameliorate worse myocardial performance and accelerate appropriate testing of therapeutic agents that can improve cardiac contractility and contraction.

## Spontaneous Ectopy

TRP channels regulate Ca^2+^-handling through interaction with Ca^2+^-dependent regulatory proteins. This interaction can lead to spontaneous ectopy, but how this may occur is not known. Spontaneous ectopy may be regarded as uncontrolled variability in heart rhythm during repolarization, which may or may not be pathological. Cycles of Ca^2+^ fluxes during normal heart beat characterize excitation- contraction coupling and permit homogenous action of cardiac sarcomeres. Increased Ca^2+^ influx, Ca^2+^ leaks, spontaneous oscillatory of Ca^2+^ and Ca^2+^ overload disturb systole and diastole and cause arrhythmias (30). As stated in previous sections, this implicates the regulation and mechanisms of Ca^2+^-handling. These processes are schematically summarized in Figure 2, organellar mechanisms of TRP channel mediated pathological Ca^2+^-handling. TRPM4 and TRPC channels trigger Ca^2+^ entry, through membrane depolarization and permeability, respectively, consequently leading to Ca^2+^ overload. Ca^2+^ overload promote alteration in action potential wave form, Ca^2+^-mishandling, inotropy, and sustain electrical remodeling, through Ca^2+^ transients. Ca^2+^ overload and large Ca^2+^ transients increase repolarization (31). Prolonged action potential duration leads to early afterdepolarizations (EAD) and delayed afterdepolarizations (DAD) ectopic firing. EADs and DADs are classes of ectopy (32).

EADs are abnormally secondary cell membrane depolarizations during repolarization phases of action potential. Prolongation of APD is the principle factor that causes EAD, because reduced repolarization enables *I*Ca,_L_ to recover from inactivation, which leads to depolarizing inward movement of Ca^2+^ ions along the plateau phase of action potential to produce Ca^2+^ inward current. EAD is also caused by increases in NCX current. DADs are also abnormally secondary cell membrane depolarizations during the repolarization phases but are known to be caused by abnormally diastolic Ca^2+^ release from sarcoplasmic reticulum Ca^2+^ stores, which NCX also contributes to. In response to transmembrane Ca^2+^ entry, a specialized Ca^2+^-dependent regulatory apparatus, SR Ca^2+^ channels known as RyRs release Ca^2+^. RyRs are closed in diastolic condition but are open if functionally defective or if the SR Ca^2+^ is above physiological arrange (33). Under physiological conditions and in healthy hearts, exercise-induced activation of sympathetic nervous system increases catecholamines produced from the chromaffin cells, which bind to and stimulate the G protein-coupled β-adrenergic receptors. This activation, in turn, stimulates adenylate cyclase to activate the cAMP-dependent protein kinase A (PKA). PKA phosphorylates major Ca^2+^-handling proteins, such PLB, RYR2, and L-type Ca^2+^ channel. Increased RyR2 Ca^2+^ release in the SR and enhanced Ca^2+^ by SERCA2a, enhances [Ca^2+^]i and contractility. PKA hyperphosphorylation of RyR2 reduces the binding affinity of the RYR2-stabilizing subunit, calstabin2, producing robust activity of the RYR2 channel to Ca^2+^-dependent activation (34). PKA hyperphosphorylation of RyR2 produces a diastolic SR Ca^2+^ leak in cardiomyocytes leading to persistently diminished SR Ca^2+^ content and contractility (34). RYR2 channel diastolic calcium leak contributes to abnormal contractility in arrhythmogenesis and sudden cardiac death (35). Furthermore, SERCA2a diminished Ca^2+^ loading and enhanced Ca^2+^ exflux through NCX also results in SR reduced Ca^2+^ loading and reduced cardiac contractility. 1 Ca^2+^ release during the diastole is exchanged for 3 extracellular Na^+^ by NCX. The result of this is a net depolarizing inward positive-ion movement called transient inward current (I_ti_) that underlies the DADs, and contractile dysfunction (36).

EADs and DADs appear to be mediated by TRPM4 protein. TRPM mediated Ca^2+^ signaling can modulate action potential wave form and cause EADs. TRPM can also modulate diastolic Ca^2+^ release from sarcoplasmic reticulum Ca^2+^ stores and cause DADs. 9-Phenanthrol, an inhibitor of TRPM4 channel abolished hypoxia and re-oxygenation-induced EADs in a mouse model (37). TRPM4-induced ectopy can be drawn on ion channel determinant of cell membrane depolarization and action potential morphology. TRPM4b a calcium activated non-selective (CAN) channel regulates cell membrane depolarization (38). TRPM4 activation is a process that contributes to the controls of the magnitude of Ca^2+^ influx by regulating membrane potential, and intracellular Ca^2+^ increased through *I*Ca,_L_ upon TRPM4 protein depletion (11).

CAN channel activity had been suggested to contribute to I_ti_ initiated by Ca^2+^ waves that underline DAD. I_ti_ has been described in Purkinje fibers, atrial and ventricular cardiomyocytes, and in sinoatrial node cells (39). In sinoatrial node (SAN), cardiac automaticity resulted from a CAN current that is attributed in part to TRPM4 (40), which slowed diastolic depolarizations lope due to the nature of “funny” current, and NCX activity (39). The molecular identity of I_ti_ is controversial, but it appears to reflect 3 Ca^2+^- dependent components: NCX, Ca^2+^-activated chloride current and current mediated by CAN channels such as TRPM4 (40). Put together, these studies support the hypothesis that I_ti_ is mediated by TRPM4. However, that I_ti_ is mediated by TRPM4 may be questioned by the fact that the single channel conductance of the I_ti_-mediating channel was 120 Ps (41), which is much larger than the ~25 pS of TRPM4 (38).

### Significance

The past few decades have witnessed tremendous evolution in characterization of the molecular and genetic mechanisms of acquired and inherited arrhythmias. The mechanisms are as numerous as the phenotypes with which the arrhythmias present. Identifying the sources that control Ca^2+^ is novel in understanding arrhythmogenesis. The TRP channels mediate Ca^2+^ flux and voltage changes across membranes. Regulation of Ca^2+^-handling by the TRP channels indicate they can potentially boost Ca^2+^ cycling disorders. Plasma membrane sensory and metabotropic TRPM4 subgroup is a drug candidate for Brugada syndrome and familial heart blocker (42). Better understanding of the TRP channels, Ca^2+^-handling and contractility is crucial.

## Conclusion

Essentially, studies illustrating the roles of TRP channels in pathological Ca^2+^-handling and spontaneous ectopy are lacking. This study reflects on TRPM and TRPC seminal works, based on their biophysical properties, to state mechanistically their potential implications in Ca^2+^ signaling dynamics. Intracellular Ca^2+^ homeostasis show tremendous changes during cardiac cycle. Intracellular Ca^2+^ homeostasis is regulated by spatial signaling processes within restricted regions, rather than by changes in global cytosolic [Ca^2+^]_i_. [Ca^2+^]_i_ is mediated by Ca^2+^ release from intracellular organelles, Ca^2+^ entry across plasma membrane through receptor agonists, receptor-activated Ca^2+^ channels, voltage-gated Ca^2+^ channels, and by ligand-gated cation channels. It appears that while overexpression of the TRPCs increase [Ca^2+^]_i_, TRPM4 depletion increases [Ca^2+^]_i_, through Ca^2+^ influx. This can be explained by the biophysical properties of the channels. Mechanization of [Ca^2+^]_i_, is defective in heart failure, failing hearts, cardiomyopathy, arrhythmia and sudden cardiac deaths. Defective intracellular Ca^2+^ homeostasis is responsible for pathological Ca^2+^-handling and contractile dysfunction. Hearts in these conditions show abnormal contractility, characterized by decreased SR Ca^2+^ sequestration, diminished intracellular Ca^2+^ transients, and enhanced diastolic SR Ca^2+^ leak activity. Sources that regulate these processes are unknown completely, and may include TRP channels and TRP channels SOCE associated mechanisms. Highlighting a potential therapeutic opportunity of the channels, this work discussed TRP channels mediated pathologic Ca^2+^-handling in spontaneous ectopy, and stated further scopes for TRP channels functions in cardiac pathophysiology.

## Author Contributions

The author confirms being the sole contributor of this work and has approved it for publication.

### Conflict of Interest Statement

The author declares that the research was conducted in the absence of any commercial or financial relationships that could be construed as a potential conflict of interest.
